# Radiomics Features of ^18^F-Fluorodeoxyglucose Positron-Emission Tomography as a Novel Prognostic Signature in Colorectal Cancer

**DOI:** 10.3390/cancers13030392

**Published:** 2021-01-21

**Authors:** Jeonghyun Kang, Jae-Hoon Lee, Hye Sun Lee, Eun-Suk Cho, Eun Jung Park, Seung Hyuk Baik, Kang Young Lee, Chihyun Park, Yunku Yeu, Jean R. Clemenceau, Sunho Park, Hongming Xu, Changjin Hong, Tae Hyun Hwang

**Affiliations:** 1 Department of Surgery, Gangnam Severance Hospital, Yonsei University College of Medicine, Seoul 06273, Korea; camp79@yuhs.ac (E.J.P.); whitenoja@yuhs.ac (S.H.B.); 2 Department of Quantitative Health Sciences, Lerner Research Institute, Cleveland Clinic, Cleveland, OH 44195, USA; chihyun@kangwon.ac.kr (C.P.); yunkuyeu@gmail.com (Y.Y.); ClemenJ@ccf.org (J.R.C.); parks@ccf.org (S.P.); xuh3@ccf.org (H.X.); hongc2@ccf.org (C.H.); 3 Department of Nuclear Medicine, Gangnam Severance Hospital, Yonsei University College of Medicine, Seoul 06273, Korea; DOCNUKE@yuhs.ac; 4 Biostatistics Collaboration Unit, Yonsei University College of Medicine, Seoul 06273, Korea; HSLEE1@yuhs.ac; 5 Department of Radiology, Gangnam Severance Hospital, Yonsei University College of Medicine, Seoul 06273, Korea; JJONDOL@yuhs.ac; 6 Department of Surgery, Severance Hospital, Yonsei University College of Medicine, Seoul 03722, Korea; kylee117@yuhs.ac; 7 Department of Computer Science and Engineering, Kangwon National University, Chuncheon-si, Gangwon-do 24341, Korea; 8 Interdisciplinary Graduate Program in Medical Bigdata Convergence, Kangwon National University, Chuncheon-si, Gangwon-do 24341, Korea

**Keywords:** colorectal cancer, ^18^F-fluorodeoxyglucose positron-emission tomography, prognosis, progression-free survival, radiomics

## Abstract

**Simple Summary:**

Currently, the optimal treatment for colorectal cancer (CRC) is planned on the basis of the results of preoperative imaging studies. Previous studies investigating the impact of radiomics signatures derived from positron-emission tomography (PET) images mainly focused on patients with rectal cancer, who underwent preoperative chemoradiotherapy, and included a relatively small number of patients, without a validation set. The impact of PET-based radiomics signature analysis in patients undergoing curative-intent radical surgery, with or without chemotherapy, has not been extensively investigated. Thus, we aimed to identify the prognostic value of radiomics signature from^18^F-fluorodeoxyglucose (^18^F-FDG) PET images by assessing the imaging features to predict the progression-free survival in patients with CRC. This study demonstrated that radiomics features derived from PET-CT images can help stratify patient prognosis and additionally increase diagnostic accuracy with respect to conventional clinicopathological data-driven prediction model in patients with CRC.

**Abstract:**

The aim of this study was to investigate the prognostic value of radiomics signatures derived from ^18^F-fluorodeoxyglucose (^18^F-FDG) positron-emission tomography (PET) in patients with colorectal cancer (CRC). From April 2008 to Jan 2014, we identified CRC patients who underwent ^18^F-FDG-PET before starting any neoadjuvant treatments and surgery. Radiomics features were extracted from the primary lesions identified on ^18^F-FDG-PET. Patients were divided into a training and validation set by random sampling. A least absolute shrinkage and selection operator Cox regression model was applied for prognostic signature building with progression-free survival (PFS) using the training set. Using the calculated radiomics score, a nomogram was developed, and its clinical utility was assessed in the validation set. A total of 381 patients with surgically resected CRC patients (training set: 228 vs. validation set: 153) were included. In the training set, a radiomics signature labeled as a rad_score was generated using two PET-derived features, such as gray-level run length matrix long-run emphasis (GLRLM_LRE) and gray-level zone length matrix short-zone low-gray-level emphasis (GLZLM_SZLGE). Patients with a high rad_score in the training and validation set had a shorter PFS. Multivariable analysis revealed that the rad_score was an independent prognostic factor in both training and validation sets. A radiomics nomogram, developed using rad_score, nodal stage, and lymphovascular invasion, showed good performance in the calibration curve and comparable predictive power with the staging system in the validation set. Textural features derived from ^18^F-FDG-PET images may enable detailed stratification of prognosis in patients with CRC.

## 1. Introduction

Colorectal cancer (CRC) is the second most common cancer in women and the third most common in men worldwide [1]. Currently, planning appropriate treatment for CRC is based on the results of preoperative imaging studies. When CRC is confirmed pathologically, usually via an endoscopic procedure, staging abdominopelvic computed tomography (CT) or chest CT is used next for detection of distant metastases. However, if systemic metastasis is definitely confirmed via an abdominopelvic or chest CT, adding ^18^F-fluorodeoxyglucose (^18^F-FDG) positron-emission tomography (PET) or ^18^F-FDG-PET/CT to aid for further clinical decision-making is controversial. Some studies have demonstrated that conventional PET-derived parameters, such as maximum standardized uptake value (SUVmax), metabolic tumor volume (MTV), and total lesion glycolysis (TLG), carry their own significance in stratifying the survival of patients with CRC [2,3,4,5]. However, other studies have yielded equivocal results [6,7]. The prognostic impact of these conventional PET-based parameters was not evident for patients with CRC, and the National Comprehensive Cancer Network guidelines recommended that ^18^F-FDG-PET or PET/CT should be used selectively for potentially surgically curable metastatic diseases or should only be used to evaluate an equivocal finding on a contrast-enhanced CT or MRI [8]. Thus, it is necessary to explore the additional clinical efficacy of PET scans in the management of CRC.

Radiomics has frequently been adopted for subtype classification, evaluation of lymph node metastasis or distant metastasis, and prediction of treatment response or prognosis in many types of cancers [9,10,11,12]. It was reported that intratumoral heterogeneity (ITH) was associated with progression and treatment response [13,14]. Although most of the tumors showed ITH [13], assessment of ITH using a single spatially biased biopsy is somewhat difficult [15]. Detection of unseen information reflecting intratumoral heterogeneity might be the main motivation for applying a radiomics approach in the oncology field [16]. Several studies have investigated the practicality of using ^18^F-FDG-PET radiomics in CRC patients. Primarily, baseline textural features, such as homogeneity, coarseness, dissimilarity, and contrast from the neighborhood intensity difference matrix, kurtosis of the absolute gradient, and coefficient of variation of SUV, have been analyzed as prognostic factors for survival and as a predictor of response for preop CRT in patients with rectal cancer [17,18,19,20]. Nevertheless, the application of baseline 18F-FDG-PET signature as a prognostic factor in CRC patients who underwent curative intent resection followed by selective chemotherapy has been limitedly reported.

Thus, we aimed to identify quantitative ^18^F-FDG-PET-based imaging biomarkers, using a radiomics approach, to predict survival outcomes in patients with CRC.

## 2. Materials and Methods

### 2.1. Study Population

We retrospectively reviewed clinical information and radiological images of patients with CRC who underwent curative intent resection at Gangnam Severance Hospital, Yonsei University College of Medicine, between April 2008 and January 2014. Patients with CRC who underwent ^18^F-FDG-PET as a preoperative diagnostic modality were initially selected. Inclusion criteria included the following: biopsy-proven primary CRC, ^18^F-FDG-PET used as the initial baseline diagnostic tool before starting any neoadjuvant treatment and within 2 months of surgery, and the availability of follow-up data and clinical information. Exclusion criteria were PET images unsuitable for further analyses, PET images taken outside of our hospital, and the presence of a lesion that was not identifiable or features that were not extractable from PET images. Patients whose metabolic tumor volume was less than 5.0 mL were also excluded because these lesions could be affected by partial volume effects [21,22]. A total of 381 individuals (224 men; age: 22–88 years) who had stage I–IV CRC and underwent surgery were included in this study (Figure S1).

All procedures performed in studies involving human participants were in accordance with the ethical standards of the institutional and/or national research committee and with the 1964 Helsinki declaration and its later amendments or comparable ethical standards.

### 2.2. PET/CT Protocol

All patients fasted for at least 6 h before the PET/CT scan and had blood glucose levels less than 140 mg/dL before intravenous administration of ^18^F-FDG (5.5 MBq/kg of body weight). Sixty minutes after intravenous administration of ^18^F-FDG, PET/CT scans were performed with a hybrid PET/CT scanner (Biograph 40 TruePoint or Biograph mCT 64, Siemens Healthcare Solutions USA, Inc., Knoxville, TN, USA). Whole-body CT images were obtained first for attenuation correction using automatic dose modulation with a reference of 40 mA and 120 kV without contrast enhancement. Afterward, PET data were acquired from the skull base to the proximal thigh for 3 min per bed position in three-dimensional mode. Positron-emission tomography images from the mCT 64 scanner were reconstructed using the ordered-subset expectation maximization (OSEM) algorithm with the point spread function (PSF), time of flight (TOF) modeling, and a 5 mm Gaussian post-filtering process (21 subsets and two iterations). For reconstructing the PET images from the Biograph 40 scanner, only the OSEM algorithm with a 5 mm Gaussian post-filtering (eight subsets and four iterations) was used. Through regular standardization and using a phantom for quality assurance, we minimized differences in the measurements of the standardized uptake value (SUV) between the two scanners to less than 10%.

### 2.3. Feature Extraction for Radiomics Analysis

A primary volume of interest (VOI) of ^18^F-FDG-PET was manually drawn around the tumor, avoiding physiological FDG uptake, particularly at both urinary tracts. In cases of patients with stage IV CRC, the primary lesion of the colon or rectum was considered rather than the metastatic foci. The relevance of these manually delineated initial VOIs was assessed before performing further automatic segmentation. The final VOI of the primary tumor lesion was automatically defined on PET images with a threshold of 40% of the SUVmax.

A total of 47 quantitative features from VOIs of each patient’s PET image were extracted using the LIFEx software, which is currently an open-source software (http://www.lifexsoft.org) [23]. Algorithms used to obtain histogram-based, shape and size, and second and high-order features are illustrated in Tables S1–S6 (Supplementary Materials). Conventional parameters, such as SUVmax, SUVmean, and MTV were measured from the final VOI. Total lesion glycolysis was calculated as SUVmean × MTV.

Initially, the interobserver agreement for the features extracted by a nuclear medicine board-certified physician and a single trained physician, both blinded to the patients’ clinical outcomes, was measured using a sample of 43 patients randomly selected from our cohort. After comparing the intraclass correlation coefficient (ICC) for these two readers, further measurements of the remaining cases were done by a single trained physician (Table S7).

### 2.4. Feature Selection, Building of Rad_Score, and Validation

Patients were allocated to training and validation sets using random sampling at a fixed ratio; 60% of patients were assigned to the training set and the remaining 40% were assigned to the validation set. The percentages were selected by considering the number of events in each group.

The least absolute shrinkage and selection operator (LASSO) algorithm, in the context of a Cox model, was applied to implement a meaningful feature selection scheme, using the association of every feature with progression-free survival (PFS) of patients in the training set [24]. Since the values obtained in the initial stage had diverse ranges, we standardized the feature values for the LASSO regression (Figure S2). Thus, a combination of feature variables, called the rad_score, could be generated. The steps for extracting those features used in the rad_score calculation are explained in the Supplementary Materials. The “glmnet” package was used to perform the LASSO Cox regression analysis.

After generating the rad_score using the LASSO Cox regression model in the training set, the optimum cutoff point of the rad_score was selected on the basis of the association with PFS of patients in the training set, using X-tile software (Version 3.6.1, Yale University School of Medicine, New Haven, CT, USA) [25]. The optimal cutoff value was defined as the value that produced the largest χ^2^ in the Mantel–Cox test, and patients were divided into the high- and low-risk subgroups on the basis of this value. This cutoff value for the rad_score in the training set was applied to the validation set to explore the potential association of the rad_score with PFS. A multivariable Cox proportional hazards model was applied to identify the potential of the rad_score as an independent prognostic biomarker in the training set, the validation set, and the overall (training + validation) set.

### 2.5. Development and Validation of the Radiomics Nomogram

To confirm the prognostic impact of using the rad_score in a clinical setting, the radiomics nomogram was generated using the training set, and then validated in the validation set. The radiomics nomogram was composed of the radiomics signature and independent clinicopathologic predictors, according to the multivariable Cox regression analysis. The Akaike information criterion (AIC) and Harrell’s concordance index (C-index) were used to compare the performance between the radiomics nomogram and the American Joint Committee on Cancer (AJCC) stage in the training set, the validation set, and the overall set.

Calibration curves, which compared the predicted survival with the actual survival, were generated to explore the performance characteristics of the developed nomogram in the validation set.

### 2.6. Statistical Analysis

All statistical analyses were performed using SPSS software, version 23.0 (SPSS, Chicago, IL, USA) and R version 3.5.1 (R-project, Institute for Statistics and Mathematics, Vienna, Austria). Continuous data were described as the mean ± standard deviation and were analyzed using the Student’s *t*-test or Wilcoxon rank sum test. Categorical data were analyzed using Pearson’s chi-squared test or Fisher’s exact test for dichotomous parameters.

All radiomics features obtained from baseline PET examination were normalized by transforming the data into new scores with a mean of 0 and a standard deviation of 1. Progression-free survival was defined from the date of surgery until the date of recurrence detection, last follow-up, or death. The patients alive at the last follow-up were censored. The Kaplan–Meier method was used to construct survival curves, and the log-rank test was used to compare survival rates between groups.

A univariable analysis was performed to calculate the hazard ratio of the single variables in the Cox proportional hazards model after entering one of the variables under investigation in the calculation model. After calculating all hazard ratios in the univariable analysis, the parameters that showed statistical significance (*p* < 0.05) or potential significance (*p* < 0.1) were further used in the multivariable analysis. Multivariable survival analyses with forward stepwise selection were performed using the Cox proportional hazards model to test for independent significance of different factors.

The coefficients of the multivariable Cox regression model in the training set were used to construct a nomogram with the “rms” package of the R software. The calibration curves were used to evaluate the clinical usefulness of the nomogram. The AIC and C-index were calculated to compare the radiomics nomogram and AJCC stage. A smaller AIC value indicated a better goodness of fit for predicting outcomes and a higher C-index value indicated a better concordance of survival times [26,27]. A two-sided *p* < 0.05 was considered statistically significant.

## 3. Results

### 3.1. Patient Characteristics

A total of 381 patients were included in our analysis. There were 43 recurrences reported after a mean follow-up period of 36.4 months (interquartile range; 27–60 months). Our initial cohort was categorized into two groups by random sampling at a fixed ratio; 228 patients (60%) were assigned to the training set and the remaining 153 patients (40%) were assigned to the validation set. There were 25 and 18 recurrences in the training and validation sets, respectively.

Patient characteristics in the training and validation sets are shown in Table 1. Except for the lymphovascular invasion (LVI) rate, there were no statistically significant differences observed in the clinicopathologic findings between the training set and the validation set. There was no significant difference in the mean and standard deviation of the rad_score between the two groups.

Analysis of the interobserver agreement of the features extracted from PET images yielded a mean ICC value, for the 47 radiomics features, of 0.932 (range 0.78–0.99) (Table S7, Supplementary Materials).

### 3.2. Radiomics Signature-Based Prediction Model

Two features, gray-level run length matrix long-run emphasis (GLRLM_LRE) and gray-level zone length matrix short-zone low-gray-level emphasis (GLZLM_SZLGE), with coefficients of 0.07079258 and 0.11149516, respectively, were selected in the LASSO Cox regression model. The rad_score was defined as 0.07079258 × GLRLM_LRE + 0.11149516 × GLZLM_SZLGE (Figure S3).

The median rad_score was −0.0495 (range −0.1394 to 1.4635). The optimum cutoff value generated by the X-tile program was 0.07 (Figure S4). Using this value, patients were classified into a high-risk group (rad_score ≥ 0.07) and a low-risk group (rad_score < 0.07). In the training set, the rates of histological grade 3 and mucinous type were significantly higher in the high-risk group than in the low-risk group. The rate of rectal cancer in the high-risk group was marginally higher than that in the low-risk group, but the difference did not reach statistical significance (*p* = 0.065). There was no difference in pT, pN, and AJCC stage between the high- and the low-risk groups (Table 2).

**Figure 1:**
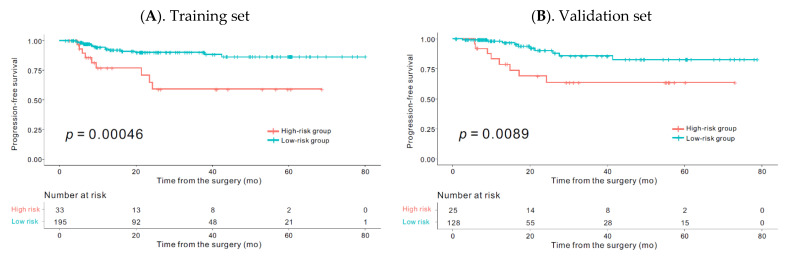
The Kaplan–Meier plot in the training and the validation set according to the high-risk and low-risk groups. Kaplan–Meier survival analyses according to the rad_score for patients in the training (**A**) and the validation (**B**) sets. The Kaplan–Meier curve showed that the radiomics signature was significantly associated with progression-free survival (PFS) in the training set (*p* < 0.001) and in the validation set (*p* = 0.008).

The Kaplan–Meier curve showed that the radiomics signature was significantly associated with PFS in the training set (*p* < 0.001) and in the validation set (*p* = 0.008) (Figure 1).

Univariable analysis demonstrated that LVI, pN, AJCC stage, and rad_score were significantly associated with PFS (all *p* < 0.05) (Table 3), while complications (*p* = 0.055), histological grade (*p* = 0.08), and pT (*p* = 0.052) showed marginal significance. Variables with *p* < 0.1 in the univariable analysis (LVI, pN, AJCC stage, rad_score, complications, histological grade, and pT) were altogether entered into the multivariable Cox proportional hazards model.

In the multivariable analysis with forward selection, presence of LVI (hazard ratio (HR) 3.73; 95% confidence interval (CI), 1.63–8.47; *p* = 0.001), pathological node positivity (HR 2.52; 95% CI, 1.01–6.26; *p* = 0.046), and a high rad_score (HR 7.82; 95% CI, 2.36–25.85; *p* < 0.001) remained independent predictors of a worse prognosis in the training set. The rad_score was also identified as an independent prognostic factor in the validation and the overall set (Table 4).

### 3.3. Calibration and Discriminative Performance Measurement of the Radiomics Nomogram

**Figure 2:**
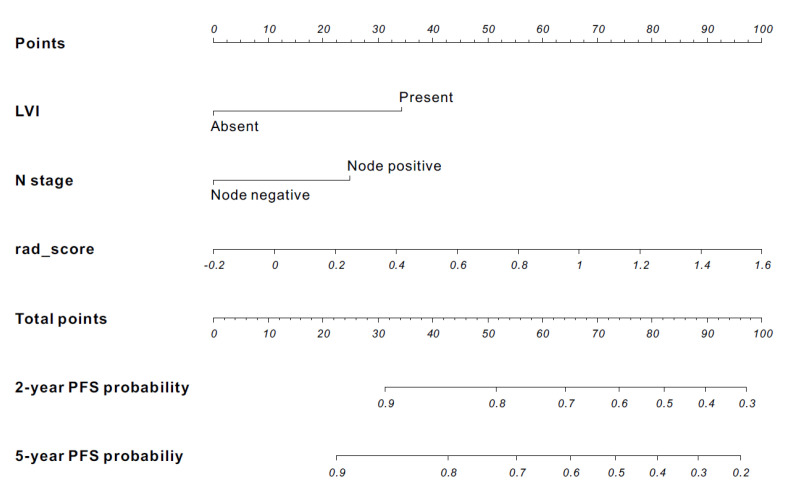
Developed nomogram to predict survival using the training set. Drawing a vertical line to the points’ axis from specific variable could determine how many points toward the probability of PFS the patient receives. The process was repeated for each variable, such as LVI, N stage, and rad_score. The points for each of the risk factors were added. The final total was then located on the total points axis.

A radiomics nomogram was established using three variables selected in the stepwise multivariable analysis in the training set (Figure 2).

Table 5 shows the comparison of the C-index and AIC between the radiomics nomogram and the AJCC stage. In the validation set, the radiomics nomogram showed a higher fitness for AIC than that in the AJCC stage (154.19 in model 4 vs. 156.861 in model 3). Radiomics nomogram and the AJCC stage showed a similar predictive power of C-index after 1000 times bootstrapping of the data (0.715; 95% CI, 0.561–0.874 in model 4 vs. 0.62; 95% CI, 0.516–0.705 in model 3; *p* = 0.101).

**Figure 3:**
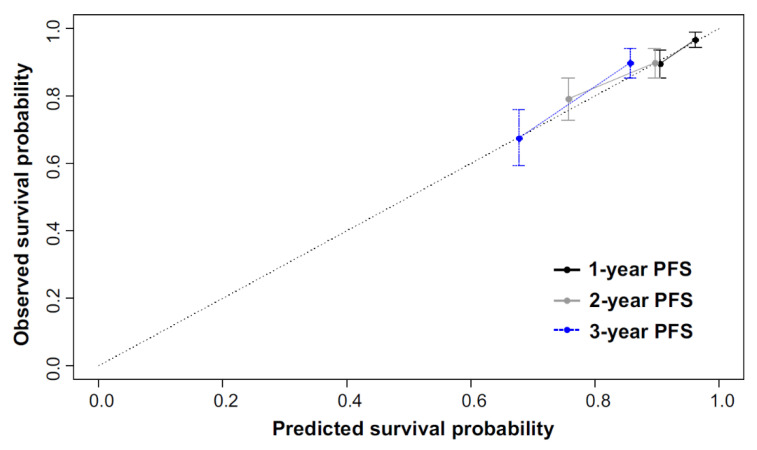
Calibration curve of radiomics nomogram in the validation set. Calibration curves for the radiomics nomograms of progression-free survival in the validation set for agreement between the estimated and the observed 1, 2 and 3 year PFS. Nomogram-estimated PFS is plotted on the *x*-axis; the observed PFS is plotted on the *y*-axis. The validation set was divided into two groups according to the survival duration; nomogram-estimated PFS and observed PFS based on the 1, 2, and 3 year PFS were calculated in each group. The diagonal dotted line represents the optimal estimation of PFS by an ideal model, in which the estimated outcome perfectly corresponds to the actual outcome. The solid lines with black, gray, and blue colors represent the performance of the nomogram. A close alignment with the diagonal dotted line indicates better estimation.

The calibration curve of the radiomics nomogram for estimating PFS showed good agreement between prediction and observation in the validation set (Figure 3).

### 3.4. Comparison of Survival within the Same Stages According to the Rad_Score

**Figure 4:**
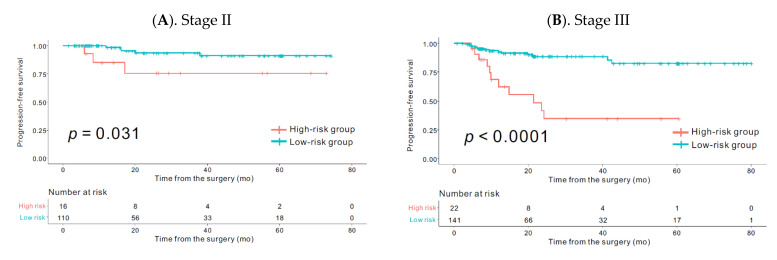
Comparison of progression-free survival of the high- and low-risk groups defined by the radiomics signature according to the stages in the overall (training + validation) set. Kaplan–Meier survival analyses according to the radiomics signature (high-risk group vs. low-risk group) in stage II (**A**) and stage III (**B**). The Kaplan–Meier curve showed that the radiomics signature was significantly associated with PFS in stage II (*p* = 0.031) and stage III patients (*p* < 0.0001) respectively.

The Kaplan–Meier curves for the subgroups stratified according to the radiomics signature (high-risk group vs. low-risk group) revealed significantly poor PFS in stage II (*p* = 0.031) and stage III (*p* < 0.0001) high-risk group patients, respectively (Figure 4).

### 3.5. Correlation between Rad_Score and PET Derived Conventional Parameters such as SUVmax, TLG, and MTV

The rad_score in overall patients was negatively correlated with SUVmax (*R* = −0.81, *p* < 0.001) and TLG (*R* = −0.37, *p* < 0.001). No significant correlation was observed between rad_score and MTV (*R* = −0.013, *p* = 0.08) (Figure S5).

## 4. Discussion

In this study, we developed and validated a radiomics nomogram using preoperative ^18^F-FDG-PET images for personalized prediction of PFS in patients with CRC. This study demonstrated that the features derived from using baseline PET could help stratify a patient’s prognosis, and the radiomics nomogram showed a comparable performance to AJCC stage for predicting PFS in patients with CRC.

Radiomics analysis with PET was recently investigated in patients with CRC. Bundschuh et al. [17] reported that the coefficient of variation of SUV correlated with PFS. Bang and colleagues [18] demonstrated that textural features, including kurtosis of the absolute gradient measured before starting preop CRT, was associated with 3 year disease-free survival (DFS). Lovinfosse and colleagues [19] revealed that homogeneity and coarseness were associated with DFS. In addition, dissimilarity and contrast from the neighborhood intensity difference matrix were correlated with overall survival. Nonetheless, previous studies that investigated the impact of radiomics derived from PET mainly focused on patients with rectal cancer, who underwent preop CRT, and included a relatively small number of patients, without using a validation set [17,18,19]. The impact of PET-based signature analysis in patients undergoing curative intent radical surgery, with or without chemotherapy, has not been extensively investigated to date.

A radiomics signature-based nomogram has been developed and validated for preoperative prediction of survival in breast cancer, gastric cancer, and early-stage non-small-cell lung cancer patients [28,29,30]. In our study, the developed nomogram incorporated a radiomics score derived from two components of the PET-based features, pN and LVI. Both are well-known clinicopathological prognostic factors in CRC patients [31,32,33]. Unexpectedly, AJCC stage was not identified as a significant factor in our multivariable Cox proportional hazards model. Our study included patients who underwent surgeries first, with curative intent, even in patients diagnosed with stage IV. Therefore, the overall prognosis of stage IV patients in this study might be better than that of commonly reported stage IV groups that included initially unresectable patients. This might be the main reason for the greater prognostic power of node positivity than stage itself, considering multicollinearity.

The radiomics signature, which was used to divide patients into high-risk and low-risk groups, was not associated with pT, pN, and AJCC stage in the training set. In addition, the patient’s PFS in stage II and III CRC could be stratified according to the radiomics signature. Collectively, these findings suggested that the radiomics signature might have different uses, compared to clinicopathological parameters, in predicting prognosis of patients with CRC, which may provide an advantage in applying this image biomarker. In the current clinical setting, estimating prognosis and using chemotherapy with an appropriate selection of chemotherapy agents are mainly dependent on the postoperative pathological stages. Wide variations in survival among patients with the same AJCC stage are already well known in patients with CRC [34,35]. Prognostic biomarkers may help select patients at high risk of recurrence, allowing customization of the follow-up monitoring process for the individual patient. The detection of circulating tumor (ct) cells or ctDNA after surgery may provide good sensitivity and specificity, although such a test would have to be compared with CEA-based detection of tumor recurrence [36,37,38,39]. Patients with a high risk of recurrence may be good candidates for liquid biopsy-based follow-up, which would be a more reasonable approach in the context of medical resource use, considering the additional cost imposed by liquid biopsies. Preoperatively, neoadjuvant chemotherapy prior to surgery has been investigated as an option for treatment, especially for locally advanced, operable colon cancer patients [40,41]. In this context, proper risk stratification prior to definite surgery may be essential in selecting candidates for neoadjuvant chemotherapy. Our study demonstrated that PET-based radiomics analysis could enhance patient stratification and, thus, can potentially guide personalized care before or after surgery in CRC patients. However, the contribution of this approach would need to be validated in future.

There were several limitations in the present study. Placement of the VOI was done by manual selection of the entire lesion, which was a time-consuming and labor-intensive task in some cases. Although our study revealed that the combination of two baseline features derived from PET could be used as a useful image biomarker, the biological underpinnings of this correlation were not evident [42]. Moreover, previously identified PET-derived radiomics features, such as coefficient of variation of SUV, kurtosis of the absolute gradient, homogeneity and coarseness, and the dissimilarity and contrast from the neighborhood intensity difference matrix, which have been proven to be important prognostic factors especially in patients with rectal cancer, were not reproducible in our radiomics signatures [17,18,19]. The discordance in the results across studies might not be easily understood and the explanation is likely to be multifactorial. First, various computer algorithms have been used for feature extraction, and the types of features extracted by each algorithm were not uniform. Moreover, we applied standardization of features as a preprocessing step before entering the data into the LASSO Cox model, following a method that has already been adopted for radiomics analysis in patients with breast or gastric cancer [28,30]. However, most previous studies of PET-derived radiomics analysis in patients with CRC did not use standardization as a preprocessing step [17,18,19,43]. The effect of this preprocessing needs to be investigated with more clinical data. Second, accurate tumor segmentation remains challenging, and there is no standard or recommended method even for radiomics analysis of PET, although the ICC value was relatively high in our study. Third, several studies have attempted to determine relatively robust features that could minimize radiomics feature variations using PET imaging [44,45,46]. Although robust feature identification and standardization might be essential, the results of those studies are also somewhat inconsistent. Different settings used for the evaluation of the radiomics features, such as using phantom vs. real patient data, absence of features with the same names, and different sources of variability and statistical assessment of robustness, make it difficult to compare the results between studies [46]. Lastly, the differences in imaging protocols of different PET/CT systems might potentially hinder the robustness of feature extractions. Therefore, for greater acceptance of radiomics in clinical decision-making, correlation and correspondence across different imaging protocol is an important step. We considered that all these situations might be common limitations in the overall application of PET-derived radiomics in oncology field. Due to the retrospective design of the study, genomic data, such as microsatellite instability (MSI) or KRAS data, were not available for all patients and, thus, could not be included in the multivariable analysis as an important genetic biomarker. However, the prognostic or predictive value of these genetic mutations in CRC has previously shown some contradictory results [47]. It may be difficult to generalize our results because the study was performed in a single institution and included a relatively small number of patients, using only an internal validation set. In addition, although our radiomics nomogram showed good calibration in the validation set, this did not improve the predictive accuracy for survival as compared to the AJCC stage. Thus, we believe that our newly developed radiomics nomogram cannot replace the currently used staging-based strategy; however, our radiomics nomogram can be used to effectively discriminate a patient’s survival within the same stage of CRC. Although further studies may be required to confirm our results, this study could be used as a proof of concept for the potential use of this approach in clinical practice.

## 5. Conclusions

In conclusion, texture analysis from PET images yields prognostic information about PFS in CRC patients. Assessing these radiomics data during the baseline diagnostic stage may assist in predicting long-term outcomes. The reproducibility of our feature-based prediction model should be evaluated in further large-scale studies.

## Figures and Tables

**Table 1:** Comparison of patients’ demographics between the training set and the validation set.

Variables	Subcategory	Training Set (*n* = 228) *n* (%)	Validation Set (*n* = 153) *n* (%)	*p*
Sex	Male	132 (57.9)	92 (60.1)	0.743
	Female	96 (42.1)	61 (39.9)	
Age (years)	<70	156 (68.4)	107 (69.9)	0.841
	≥70	72 (31.6)	46 (30.1)	
ASA	1	110 (48.2)	72 (47.1)	0.163
	2	88 (38.6)	62 (40.5)	
	3	30 (13.2)	19 (12.4)	
BMI (kg/m^2^)	<25	161 (70.6)	115 (75.2)	0.391
	≥25	67 (29.4)	38 (24.8)	
Preop CEA (ng/mL)	<5	147 (64.5)	100 (65.4)	0.946
	≥5	81 (35.5)	53 (34.6)	
Tumor location	Colon	165 (72.4)	117 (76.5)	0.438
	Rectum	63 (27.6)	36 (23.5)	
Complications	No	178 (78.1)	127 (83)	0.293
	Yes	50 (21.9)	26 (17)	
Histologic grade	G1	23 (10.1)	14 (9.2)	0.936
	G2	186 (81.6)	127 (83)	
	G3 and Mucinous	19 (8.3)	12 (7.8)	
LVI	Absent	175 (76.8)	98 (64.1)	0.010
	Present	53 (23.2)	55 (35.9)	
No. of Retrieved LNs	(Mean ± SD)	26.7 ± 16.7	25.5 ± 16.7	0.495
LN numbers	<12	25 (11)	20 (13.1)	0.644
	≥12	203 (89)	133 (86.9)	
pT ^a^	T1–T2	39 (17.1)	24 (15.7)	0.504
	T3	156 (68.4)	100 (65.4)	
	T4	33 (14.5)	29 (19)	
pN ^b^	Negative	113 (49.6)	64 (41.8)	0.168
	Positive	115 (50.4)	89 (58.2)	
AJCC Stage ^c^	I	30 (13.2)	14 (9.2)	0.626
	II	77 (33.8)	50 (32.7)	
	III	93 (40.8)	69 (45.1)	
	IV	28 (12.3)	20 (13.1)	
Distant metastasis	No	200 (87.7)	133 (86.9)	0.944
	Yes	28 (12.3)	20 (13.1)	
MSI	MSS/MSI-Low	138 (60.5)	102 (66.7)	0.371
	MSI-High	15 (6.6)	11 (7.2)	
	No data	75 (32.9)	40 (26.1)	
KRAS	Wild	72 (31.6)	53 (34.6)	0.794
	Mutant	35 (15.4)	21 (13.7)	
	No data	121 (53.1)	79 (51.6)	
Postoperative chemotherapy	No	84 (36.8)	61 (39.9)	0.625
	Yes	144 (63.2)	92 (60.1)	
Radiotherapy	No	212 (93)	143 (93.5)	>0.99
	Preoperative or postoperative	16 (7)	10 (6.5)	
rad_score	(Mean ± SD)	0.0 ± 0.2	0.0 ± 0.1	0.867 ^d^

Abbreviations—SD: standard deviation; ASA: American Society of Anesthesiology; BMI: body mass index; CEA: carcinoembryonic antigen; LVI: lymphovascular invasion; LN: lymph node; MSI: microsatellite instability; MSS: microsatellite Stable; KRAS: v-Ki-ras2 Kirsten rat sarcoma viral oncogene homolog. ^a^ Patients who underwent chemoradiotherapy before surgery were ypT stage. Patients with pathologic complete response in rectal cancer were included in pT1 for statistical reasons. ^b^ Patients who underwent chemoradiotherapy before surgery were ypN stage. ^c^ AJCC denotes the American Joint Committee on Cancer. Patients who underwent chemoradiotherapy before surgery were yp Stage. Patients with pathologic complete response with no lymph node metastasis in rectal cancer were included in yp Stage I for statistical reasons. ^d^ Wilcoxon rank sum test.

**Table 2:** Comparison of patients’ demographics between the high-risk group and the low-risk group in the training set.

Variables	Subcategory	Low-Risk Group (*n* = 195) *n* (%)	High-Risk Group (*n* = 33) *n* (%)	*p*
Sex	Male	113 (57.9)	19 (57.6)	>0.99
	Female	82 (42.1)	14 (39.9)	
Age (years)	<70	132 (67.7)	24 (72.7)	0.709
	≥70	63 (32.3)	9 (27.3)	
ASA	1	91 (46.7)	19 (57.6)	0.482
	2	77 (39.5)	11 (33.3)	
	3	27 (13.8)	3 (9.1)	
BMI (kg/m^2^)	<25	132 (67.7)	29 (87.9)	0.032
	≥25	63 (32.3)	4 (12.1)	
Preop CEA (ng/mL)	<5	124 (63.6)	23 (69.7)	0.630
	≥5	71 (36.4)	10 (30.3)	
Tumor location	Colon	146 (74.9)	19 (57.6)	0.065
	Rectum	49 (25.1)	14 (42.4)	
Complications	No	155 (79.5)	23 (69.7)	0.303
	Yes	40 (20.5)	10 (30.3)	
Histologic grade	G1 + G2	184 (94.4)	25 (75.8)	0.001
	G3 and Mucinous	11 (5.6)	8 (24.2)	
LVI	Absent	152 (77.9)	23 (69.7)	0.415
	Present	43 (22.1)	10 (30.3)	
No. of Retrieved LNs	(Mean ± SD)	26.8 ± 16.6	26.2 ± 17.3	0.846
LN numbers	<12	18 (9.2)	7 (21.2)	0.083
	≥12	177 (90.8)	26 (78.8)	
pT ^a^	T1–T2	33 (16.9)	6 (78.8)	0.356
	T3	136 (69.7)	20 (60.6)	
	T4	26 (13.3)	7 (21.2)	
pN ^b^	Negative	98 (50.3)	15 (45.5)	0.747
	Positive	97 (49.7)	18 (54.5)	
AJCC Stage ^c^	I	26 (13.3)	4 (12.1)	0.386
	II	68 (34.9)	9 (27.3)	
	III	80 (41)	13 (39.4)	
	IV	21 (10.8)	7 (21.2)	
Distant metastasis	No	174 (89.2)	26 (78.8)	0.160
	Yes	21 (10.8)	7 (21.2)	
MSI	MSS/MSI-Low	117 (60)	21 (63.6)	0.669
	MSI-High	14 (7.2)	1 (3)	
	No data	64 (32.8)	11 (33.3)	
KRAS	Wild	63 (32.3)	9 (27.3)	0.829
	Mutant	30 (15.4)	5 (15.2)	
	No data	102 (52.3)	19 (57.6)	
Postoperative chemotherapy	No	73 (37.4)	11 (33.3)	0.797
	Yes	122 (62.6)	22 (66.7)	
Radiotherapy	No	187 (95.9)	25 (75.8)	<0.001
	Preoperative or postoperative	8 (4.1)	8 (24.2)	
rad_score	(Mean ± SD)	0.0 ± 0.0	0.3 ± 0.3	<0.001 ^d^

Abbreviations—SD: standard deviation; ASA: American Society of Anesthesiology; BMI: body mass index; CEA: carcinoembryonic antigen; LVI: lymphovascular invasion; LN: lymph node; MSI: microsatellite instability; MSS: microsatellite Stable; KRAS: v-Ki-ras2 Kirsten rat sarcoma viral oncogene homolog. ^a^ Patients who underwent chemoradiotherapy before surgery were ypT stage. Patients with pathologic complete response in rectal cancer were included in pT1 for statistical reasons. ^b^ Patients who underwent chemoradiotherapy before surgery were ypN stage. ^c^ AJCC denotes the American Joint Committee on Cancer. Patients who underwent chemoradiotherapy before surgery were yp Stage. Patients with pathologic complete response with no lymph node metastasis in rectal cancer were included in yp Stage I for statistical reason. ^d^ Wilcoxon rank sum test.

**Table 3:** Univariable analysis associated with the progression-free survival in the training set.

Variables	Subcategory	Univariable Analysis	
		HR (95% CI)	*p*
Sex	Female	Ref	
	Male	0.54 (0.24–1.2)	0.136
Age (years)	<70	Ref	
	≥70	0.88 (0.38–2.04)	0.773
ASA	1 & 2	Ref	
	3	1.51 (0.44–5.07)	0.505
BMI (kg/m^2^)	<25	Ref	
	≥25	0.52 (0.19–1.39)	0.193
Preop CEA (ng/mL)	<5	Ref	
	≥5	1.3 (0.58–2.89)	0.52
Tumor location	Colon	Ref	
	Rectum	1.87 (0.84–4.12)	0.12
Complications	No	Ref	
	Yes	2.22 (0.98–5.04)	0.055
Histologic grade	G1 and G2	Ref	
	G3 and Mucinous	2.6 (0.89–7.61)	0.08
LVI	Absent	Ref	
	Present	3.96 (1.80–8.71)	<0.001
LN numbers	<12	Ref	
	≥12	0.68 (0.25–1.84)	0.46
pT ^a^	T1–T3	Ref	
	T4	2.37 (0.98–5.67)	0.052
pN ^b^	Negative	Ref	
	Positive	2.95 (1.23–7.09)	0.015
AJCC Stage ^c^	I & II	Ref	
	III & IV	3.22 (1.28–8.1)	0.012
Distant metastasis	No	Ref	
	Yes	1.16 (0.34–3.89)	0.808
MSI	MSS/MSI-Low	Ref	
	MSI-High	4.042 × 10^−8^ (0–Inf)	0.997
	No data	1.25 (0.57–2.77)	0.571
KRAS	Wild	Ref	
	Mutant	1.84 (0.41–8.25)	0.424
	No data	1.57 (0.52–4.75)	0.419
Postoperative chemotherapy	No	Ref	
	Yes	0.84 (0.36–1.97)	0.7
rad_score ^d^	Continuous	4.91 (1.73–13.92)	0.002

Abbreviations—HR: hazard ratio; CI: confidence interval; SD: standard deviation; ASA: American Society of Anesthesiology; BMI: body mass index; CEA: carcinoembryonic antigen; LVI: lymphovascular invasion; LN: lymph node; MSI: microsatellite instability; MSS: microsatellite Stable; KRAS: v-Ki-ras2 Kirsten rat sarcoma viral oncogene homolog. ^a^ Patients who underwent chemoradiotherapy before surgery were ypT stage. Patients with pathologic complete response in rectal cancer were included in pT1 for statistical reasons. ^b^ Patients who underwent chemoradiotherapy before surgery were ypN stage. ^c^ AJCC denotes the American Joint Committee on Cancer. Patients who underwent chemoradiotherapy before surgery were yp Stage. Patients with pathologic complete response with no lymph node metastasis in rectal cancer were included in yp Stage I for statistical reasons. ^d^ Continuous variable.

**Table 4:** Multivariable analysis associated with progression-free survival in the training set, validation set, and overall set.

Variables	Subcategory	Training Set		Validation Set		Overall Set	
		HR (95% CI)	*p*	HR (95% CI)	*p*	HR (95% CI)	*p*
LVI	Absent	Ref				Ref	
	Present	3.73 (1.64–8.47)	0.001			2.37 (1.22–4.59)	0.010
pT ^a^	T1–T3			Ref		Ref	
	T4			4.33 (1.66–11.29)	0.002	2.22 (1.16–4.25)	0.016
pN ^b^	negative	Ref		Ref		Ref	
	positive	2.52 (1.01–6.26)	0.046	3.38 (0.96–11.85)	0.056	2.24 (1.05–4.80)	0.037
rad_score ^c^		7.82 (2.36–25.85)	<0.001	12.18 (2.21–66.90)	0.004	8.47 (3.21–22.34)	<0.001

Abbreviations—HR: hazard ratio; CI: confidence interval; LVI: lymphovascular invasion. ^a^ Patients who underwent chemoradiotherapy before surgery were ypT stage. Patients with pathologic complete response in rectal cancer were included in pT1 for statistical reasons. ^b^ Patients who underwent chemoradiotherapy before surgery were ypN stage. ^c^ Continuous variable.

**Table 5:** Comparison of C-index and Akaike information criterion (AIC) between radiomics nomogram and AJCC stage in the training, validation, and overall set.

Parameters	Training Set (n = 228)		Validation Set (n = 153)		Overall Set (n = 381)	
	Model 1	Model 2	Model 3	Model 4	Model 5	Model 6
Included variables	AJCC stage	LVI, pN, rad_score	AJCC stage	LVI, pN, rad_score	AJCC stage	LVI, pN, rad_score
C-index (95% CI) (bootstrapped), *p*	0.64 (0.55–0.718)	0.737 (0.63–0.844)	0.62 (0.516–0.705)	0.715 (0.561–0.874)	0.628 (0.563–0.689)	0.705 (0.619–0.788)
	*p* = 0.033		*p* = 0.101		*p* = 0.014	
AIC	241.763	230.996	156.861	154.19	455.156	439.26

Abbreviations—C-index: Harrell’s concordance index; AIC: Akaike information criterion; CI: confidence interval; LVI: lymphovascular invasion; AJCC: American Joint Committee on Cancer.

## Data Availability

The datasets generated and/or analyzed during the current study are available from the corresponding author on reasonable request pending the approval of the institution(s) and trial/study investigators who contributed to the dataset.

## Supplementary Materials

The following are available online at https://www.mdpi.com/2072-6694/13/3/392/s1: Figure S1: Patient inclusion of this study; Figure S2: Comparison of heat map of 47 features from all patients before and after standardization; Figure S3: Selection of radiomics signature in PET using LASSO Cox regression model in the training set and definition of rad_score; Figure S4: Cutoff value selection using X-tile plots of the rad_score; Figure S5: Correlation between rad_score and PET-derived conventional parameters such as SUVmax, TLG, and MTV, Table S1. Definition of radiomics features for Conventional Indices, Table S2. Definition of radiomics features for First Order Features, Table S3. Definition of radiomics features for Grey level co-occurrence matrix (GLCM), Table S4. Definition of radiomics features for Grey-Level Run Length Matrix (GLRLM), Table S5. Definition of radiomics features for Neighborhood Grey-Level Different Matrix (NGLDM), Table S6. Definition of radiomics features for Grey-Level Zone Length Matrix (GLZLM), Table S7. Intraclass correlation coefficient (ICC) results according to the each variables.
