# Supplementary material for: Radiomics Features of 18F-Fluorodeoxyglucose Positron-Emission Tomography as a Novel Prognostic Signature in Colorectal Cancer
Source: Cancers (Basel). 2021 Jan 21;13(3):392. doi: 10.3390/cancers13030392 (PMC7866240; doi:10.3390/cancers13030392)
Supplement: Supplementary file 1 [file cancers-13-00392-s001.pdf]

Supplementary Materials: Radiomics Features of ^{18}F-fluorodeoxyglucose Positron-Emission Tomography as a Novel Prognostic Signature in Colorectal Cancer

Jeonghyun Kang, Jae-Hoon Lee, Hye Sun Lee, Eun-Suk Cho, Eun Jung Park, Seung Hyuk Baik, Kang Young Lee, Chihyun Park, Yunku Yeu, Jean R. Clemenceau, Sunho Park, Hongming Xu, Changjin Hong and Tae Hyun Hwang

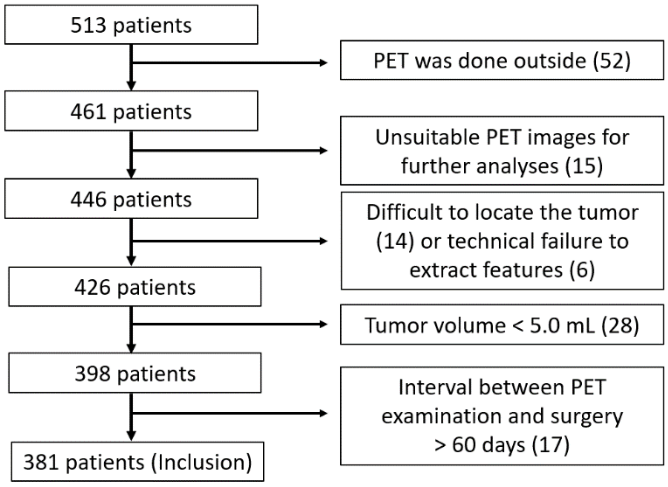

Figure S1. Patient inclusion of this study.

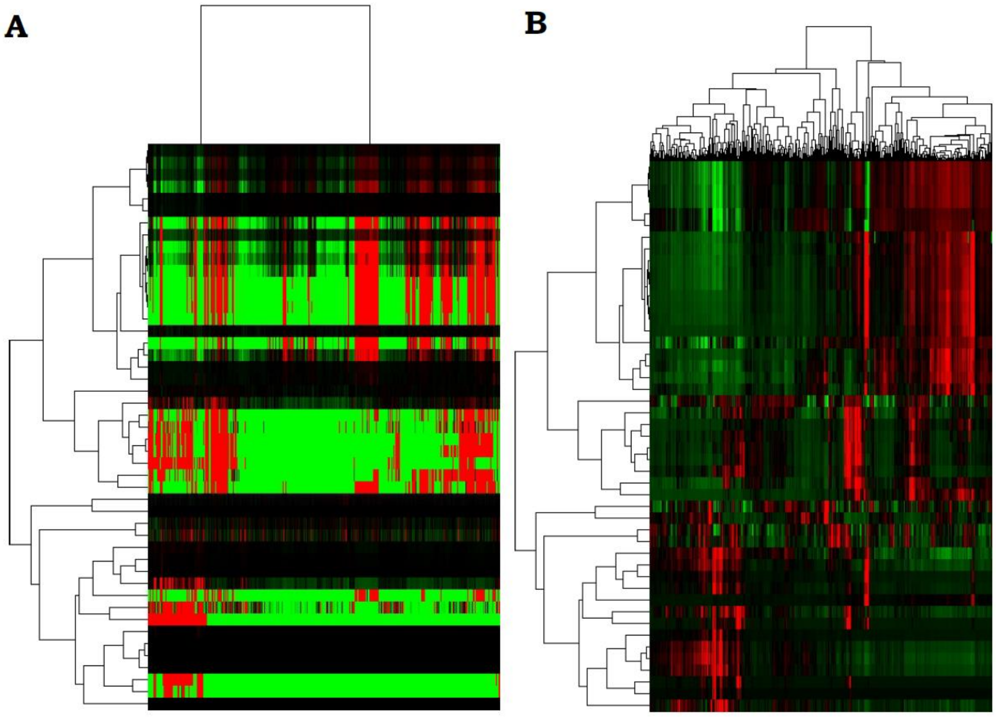

Figure S2. Comparison of heat map of 47 features from all patients before and after standardization. Graphical representation of Heat map of 47 parameters before standardization (**A**) and after standardization (**B**). Standardization is the process of putting different variables on the same scale. To standardize variables, the mean and standard deviation for a variable should be calculated. Standardized value could be defined by subtracting the mean and dividing by the standard deviation for each observed value of the variable. This process produces standard scores that represent the number of standard deviations above or below the mean that a specific observation falls.

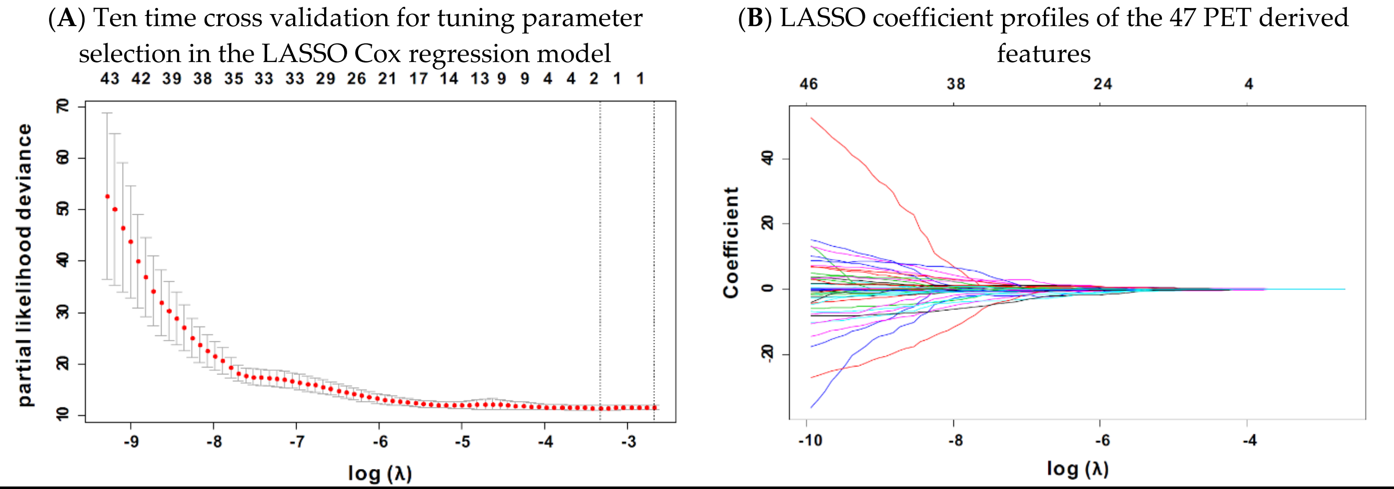

Figure S3. Selection of radiomics signature in PET using LASSO Cox regression model in the training set and definition of rad_score. The least absolute shrinkage and selection operator method (LASSO) was used for regression of high dimensional predictors. The method uses an L1 penalty to shrink some regression coefficients to exactly zero. (**A**) The partial likelihood deviance (PLD) curve was plotted versus log (λ), where λ is the tuning parameter. Solid vertical lines represent PLD ± standard error (SE). The dotted vertical lines are drawn at the optimal values by using the minimum criteria and 1-SE criteria. Tuning parameter (λ) selection in the LASSO model used 10-fold cross-validation via minimum criteria. A value λ = 0.03577575 with log (λ) = -3.330485 was chosen. (**B**) LASSO coefficient profiles of the 47 PET derived features. A coefficient profile plot was produced against the log (λ) sequence. The optimal tuning parameter resulted in two non-zero coefficients. Two features, Gray Level Run Length Matrix_Long-Run Emphasis (GLRLM_LRE) and Grey-Level Zone Length Matrix_Short-Zone Low Gray-level Emphasis (GLZLM_SZLGE), with coefficients 0.07079258, 0.11149516 respectively, were selected in the LASSO Cox regression model. The rad_score was defined as 0.07079258 × GLRLM_LRE + 0.11149516 x GLZLM_SZLGE.

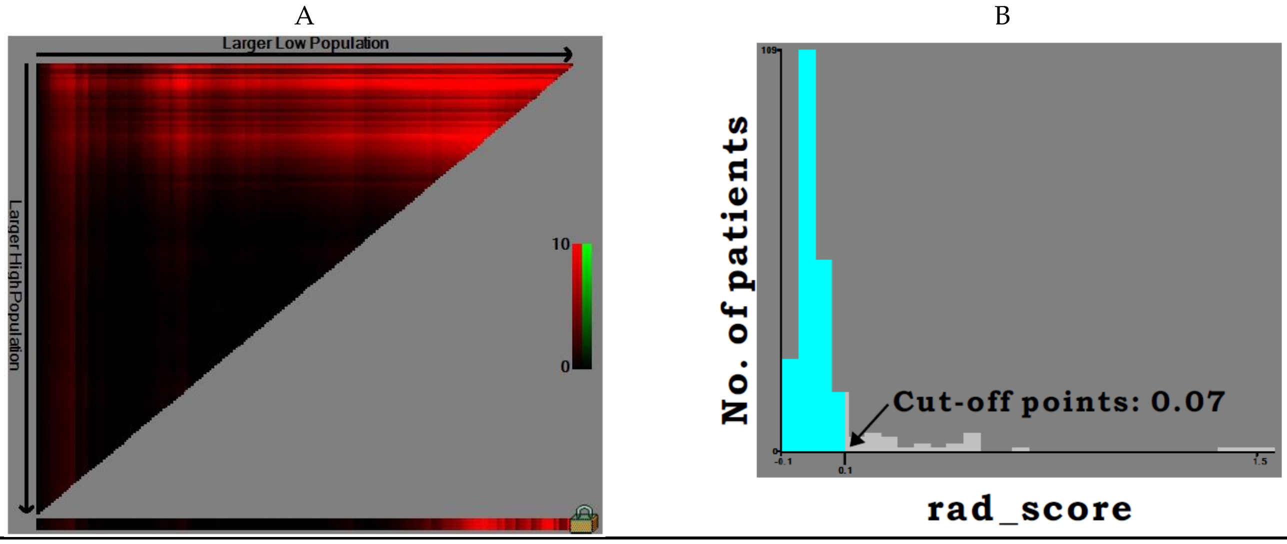

Figure S4. Cut-off value selection using X-tile plots of the rad_score. (**A**) X-tile plots of the rad_score and the points of the rad_score coloration of the plot represents the strength of the association at each division ranging from low (dark, black) to high (bright, red or green). Red represents an inverse association between the expression levels and survival of the feature, whereas green represents a direct association. (**B**) The optimal cut-off value was defined as the value that produced the largest $\chi 2$ in the Mantel-Cox test and this point was set as 0.07. Patients were divided into the high- and low-risk subgroups based on this value.

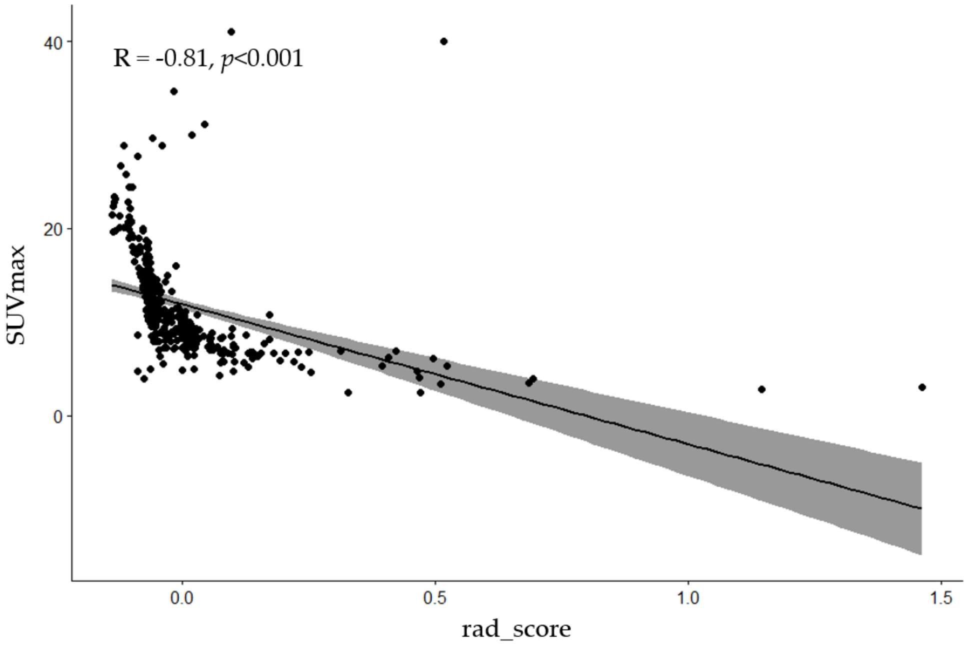

(**A**) SUVmax

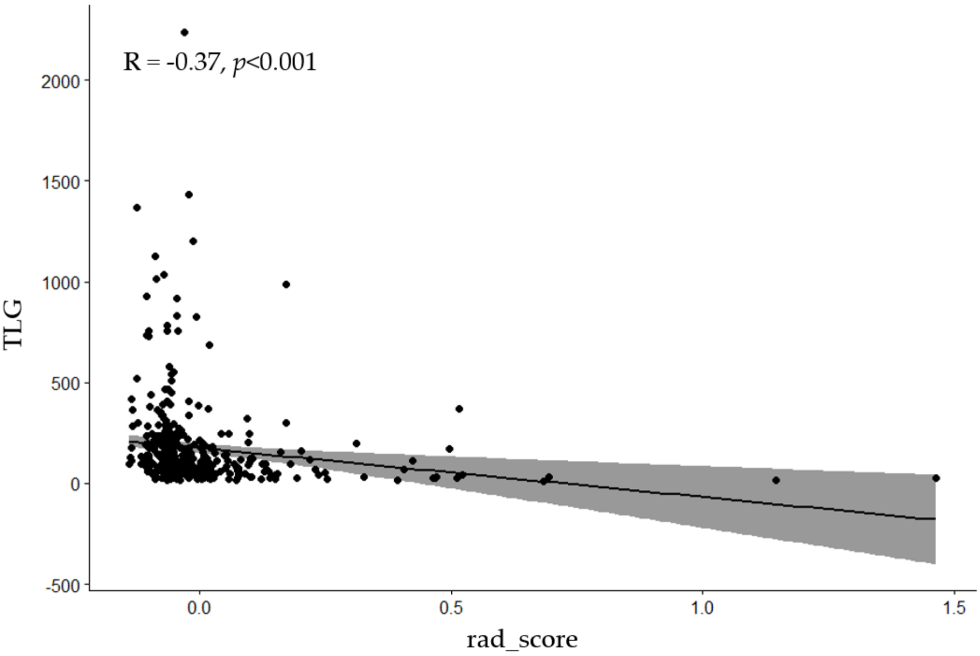

(**B**) Total lesion glycolysis (TLG)

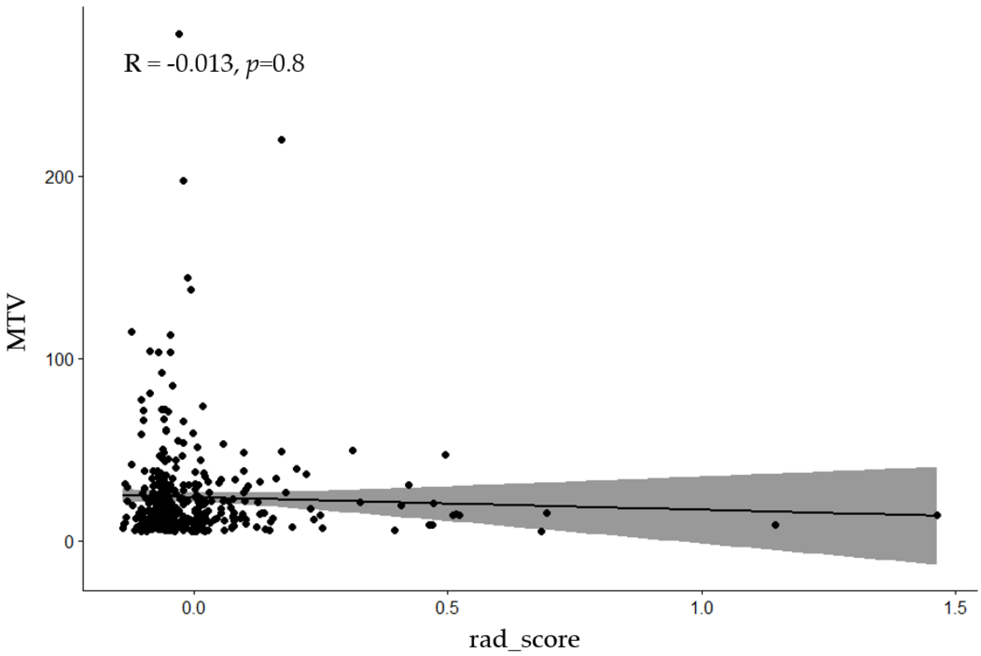

(**C**) Metabolic tumor volume (MTV)

Figure S5. Correlation between rad_score and PET derived conventional parameters such as SUVmax, TLG and MTV. (**A**) Correlation between SUVmax and rad_score. (**B**) Correlation between TLG and rad_score. (**C**) Correlation between MTV and rad_score. The relationship between variables was evaluated using the Spearman rank correlation test.

Note: These mathematic formulas used in this section are mainly derived from the website http://www.lifexsoft.org (accessed on 1 July 2019).

Table S1. Definition of radiomics features for Conventional Indices.

**Conventional Indices**
CONVENTIONAL_SUVmin
CONVENTIONAL_SUVmean
CONVENTIONAL_SUVstd
CONVENTIONAL_SUVmax
CONVENTIONAL_SUVpeak
CONVENTIONAL_TLG

CONVENTIONAL_SUVmin: minimum Standardized Uptake Value (SUV) in the volume of interest.

$$\text{CONVENTIONAL\_SUVmin} = \boldsymbol{min_i SUV_i} \quad (1)$$

CONVENTIONAL_SUVmean**:** average SUV in the volume of interest.

$$\text{CONVENTIONAL\_SUVmean} = \frac{1}{N}\sum_i \boldsymbol{SUVi} \quad (2)$$

CONVENTIONAL_SUVstd: standard deviation SUV in the volume of interest.

$$\text{CONVENTIONAL\_SUVstd} = \sqrt{\frac{1}{N}\sum_{i=1}^{N}(\boldsymbol{SUVi} - \boldsymbol{SUVmean})^2} \quad (3)$$

CONVENTIONAL_SUVmax**:** maximum SUV in the volume of interest.

$$\text{CONVENTIONAL\_SUVmax} = \boldsymbol{max_i SUV_i} \quad (4)$$

CONVENTIONAL_SUVpeak: mean of SUV in a sphere with a volume of ~1 mL and located so that the average value in the VOI is maximum.

CONVENTIONAL_TLG: the product of SUVmean by Volume in mL.

$$\text{CONVENTIONAL\_TLG} = \boldsymbol{V\,X\frac{1}{N}\sum_i SUV_i} \qquad (5)$$

Table S2. Definition of radiomics features for First Order Features.

HISTO_Skewness
HISTO_Kurtosis
HISTO_Entropy_log_{10}
HISTO_Entropy_log_2
HISTO_Energy
SHAPE_Volume_mL
SHAPE_Volume_vx
SHAPE_Sphericity
SHAPE_Compacity

HISTO_Skewness: asymmetry of the grey-level distribution in the histogram.

$$\text{HISTO\_Skewness} : \frac{\frac{1}{E}\sum_i \left(HISTO(i)-\overline{HISTO}\right)^3}{\left(\sqrt{\frac{1}{E}\sum_i \left(HISTO(i)-\overline{HISTO}\right)^2}\right)^3} \qquad (6)$$

where HISTO (i) corresponds to the number of voxels with intensity i, E is the total number of voxels in the VOI and $\overline{HISTO}$ is the average of grey-levels in the histogram.

HISTO_Kurtosis: shape of the grey-level distribution (peaked or flat) relative to a normal distribution.

$$\text{HISTO\_Kurtosis} = \frac{\frac{1}{E}\sum_i \left(HISTO(i)-\overline{HISTO}\right)^4}{\left(\frac{1}{E}\sum_i \left(HISTO(i)-\overline{HISTO}\right)^2\right)^2} \qquad (7)$$

where HISTO(i) corresponds to the number of voxels with intensity i, E the total number of voxels in the VOI and $\overline{HISTO}$ the average of grey-levels in the histogram.

HISTO_Entropy_log$_{10}$: the randomness of the distribution.

$$\text{HISTO\_Entropy}_{\_log10} = -\sum_i \boldsymbol{p(i)\cdot log10(p(i)+\varepsilon)} \qquad (8)$$

where *p(i)* is the probability of occurrence of voxels with intensity *i* and *ε*=2e-16

HISTO_Entropy_log$_2$: the randomness of the distribution.

$$\textit{HISTO\_Entropy}_{\_log2} = -\sum_i \boldsymbol{p(i)\cdot log2(p(i)+\varepsilon)} \qquad (9)$$

where *p(i)* is the probability of occurrence of voxels with intensity i and *ε*=2e-16

HISTO_Energy: the uniformity of the distribution.

$$\textit{HISTO\_Energy} = \sum_i \boldsymbol{p(i)}^2 \qquad (10)$$

SHAPE_Volume (mL and voxels): the volume of interest in mL and in voxels.

$$\textit{SHAPE\_Volume} = \sum_i \boldsymbol{Vi} \qquad (11)$$

*w*here *Vi* correspond to the volume of voxel *i* of the VOI.

SHAPE_Sphericity: how spherical a volume of interest is. Sphericity is equal to 1 for a perfect sphere.

$$\textit{SHAPE\_Sphericity} = \frac{\pi^{1/3}\cdot(6V)^{2/3}}{A} \qquad (12)$$

where V and A correspond to the volume and the surface of VOI based on the Delaunay triangulation.

SHAPE_Compacity: how compact the volume of interest is.

$$SHAPE\_Compacity = \frac{A^{3/2}}{V} \quad (13)$$

where V and A correspond to the volume and the surface of the VOI based on the Delaunay triangulation.

Table S3. Definition of radiomics features for Grey level co-occurrence matrix (GLCM).

GLCM_Homogeneity.
GLCM_Energy
GLCM_Contrast
GLCM_Correlation
$GLCM\_Entropy\_log_{10}$
$GLCM\_Entropy\_log_{2}$
GLCM_Dissimilarity

The GLCM takes into account the arrangements of pairs of voxels to calculate textural indices. The GLCM is calculated from 13 different directions in 3D with aδ-voxel distance ($\|\vec{d}\|$ relationship between neighboured voxels. The index value is the average of the index over the 13 directions in space (X, Y, Z). Seven textural indices can be computed from this matrix. An entry *(i,j)* of GLCM for one direction is equal to:

$$GLCM\triangle x \triangle y(i,j) = \frac{1}{Pairs_{ROI}} \sum_{p=1}^{N-\triangle x} \sum_{q=1}^{M-\triangle y} \begin{cases} 1\ if (I(p,q) = i, I(p+\triangle x, q+\triangle y) = j) \\ \ and\ I(p,q), I(p+\triangle x, q+\triangle y) \in ROI \\ 0\ otherwise \end{cases} \quad (14)$$

where *I(p,q)* corresponds to voxel *(p,q)* in an image *(I)* of size N∗M. The vector $\underset{d}{\rightarrow} = (\triangle x, \triangle y)$covers the 4 directions (D1, D2, D3, D4) in 2D space or 13 directions (D1, D2, ..., D13) in 3D space and $Pairs_{ROI}$. Corresponds to the number of all voxel pairs belonging to the region of interest. The GLCM reflects the distribution of co-occurring pixel values at a given offset.

GLCM_Homogeneity: the homogeneity of grey-level voxel pairs.

$$GLCM\_Homogeneity = Average\ over\ 13(or\ 4)\ directions\ (\sum_i \sum_j \frac{GLCM(i,j)}{1+|i-j|}) \quad (15)$$

GLCM_Energy: also called uniformity or second angular moment, the uniformity of grey-level voxel pairs.

$$GLCM\_Energy = Average\ over\ 13\ (or\ 4)\ directions\ (\sum_i \sum_j GLCM(i,j)^2) \quad (16)$$

GLCM_Contrast: also called Variance or Inertia, the local variations in the GLCM.

$$GLCM\_Contrast = Average\ over\ 13\ (or\ 4)\ directions\ (\sum_i \sum_j (i-j)^2 \cdot GLCM(i,j)) \quad (17)$$

GLCM_Correlation: the linear dependency of grey-levels in GLCM.

$$GLCM\_Correlation = Average\ over\ 13\ (or\ 4)\ directions\ (\sum_i \sum_j \frac{(i-\mu_i) \cdot (j-\mu_j) \cdot GLCM(i,j)}{\sigma_i \cdot \sigma_j} \quad (18)$$

where μ_i or μ_j corresponds to the average on row *i* or column *j* and σ_i and σ_j correspond to the variance on row *i* or column *j*.

$GLCM\_Entropy\_log_{10}$: the randomness of grey-level voxel pairs.

$$GLCM\_Entropy_{log10} = Average\ over\ 13(or\ 4)\ directions\ (-\sum_i \sum_j GLCM(i,j) \cdot log_{10}(GLCM(i,j) + \varepsilon)) \quad (19)$$

where ε= 2e-16

$GLCM\_Entropy\_log_{2}$: the randomness of grey-level voxel pairs.

$$GLCM\_Entropy_{log2} = Average\ over\ 13\ (or\ 4)\ directions\ \left(\sum_i \sum_j GLCM(i,j) \cdot log_2(GLCM(i,j) + \varepsilon)\right), \text{ where } \varepsilon = 2e\text{-}16 \quad (20)$$

GLCM_Dissimilarity: the variation of grey-level voxel pairs.

$$GLCM\_Dissimilarity = Average\ over\ 13\ (or\ 4)\ directions\ \left(\sum_i \sum_j |i - j| \cdot GLCM(i,j)\right) \quad (21)$$

Table S4. Definition of radiomics features for Grey-Level Run Length Matrix (GLRLM).

GLRLM_SRE : Short-Run Emphasis
GLRLM_LRE : Long-Run Emphasis
GLRLM_LGRE : Low Gray-level Run Emphasis
GLRLM_HGRE : High Gray-level Run Emphasis
GLRLM_SRLGE : Short-Run Low Gray-level Emphasis
GLRLM_SRHGE : Short-Run High Gray-level Emphasis
GLRLM_LRLGE : Long-Run Low Gray-level Emphasis
GLRLM_LRHGE : Long-Run High Gray-level Emphasis
GLRLM_GLNUr : Gray-Level Non-Uniformity for run
GLRLM_RLNU : Run Length Non-Uniformity
GLRLM_RP : Run Percentage

The GLRLM gives the size of homogeneous runs for each grey level. This matrix is computed for the 13 different directions in 3D (4 in 2D) and for each of the 11 texture indices derived from this matrix, the 3D value is the average over the 13 directions in 3D (4 in 2D). The element *(i,j)* of GLRLM corresponds to the number of homogeneous runs of *j* voxels with intensity *i* in an image and is called *GLRLM(i,j)* thereafter.

GLRLM_SRE, GLRLM_LRE: the distribution of the short or the long homogeneous runs in an image.

$$GLRLM\_SRE = Average\ over\ 13\ (or\ 4)\ directions\ \left(\frac{1}{H} \sum_i \sum_j \frac{GLRLM(i,j)}{j^2}\right) \quad (22)$$

$$GLRLM\_LRE = Average\ over\ 13\ (or\ 4)\ directions\ \left(\frac{1}{H} \sum_i \sum_j GLRLM(i,j) \cdot j^2\right) \quad (23)$$

where H corresponds to the number of homogeneous runs in the volume of interest.

GLRLM_LGRE, GLRLM_HGRE: the distribution of the low or high grey-level runs.

$$GLRLM\_LGRE = Average\ over\ 13\ (or\ 4)\ directions\ \left(\frac{1}{H} \sum_i \sum_j \frac{GLRLM(i,j)}{i^2}\right) \quad (24)$$

$$GLRLM\_HGRE = Average\ over\ 13\ (or\ 4)\ directions\ \left(\frac{1}{H} \sum_i \sum_j GLRLM(i,j) \cdot i^2\right) \quad (25)$$

GLRLM_SRLGE, GLRLM_SRHGE: the distribution of the short homogeneous runs with low or high grey-levels.

$$GLRLM\_SRLGE = Average\ over\ 13\ (or\ 4)\ directions\ \left(\frac{1}{H} \sum_i \sum_j \frac{GLRLM(i,j)}{i^2 j^2}\right) \quad (26)$$

$$GLRLM\_SRHGE = Average\ over\ 13\ (or\ 4)\ directions\ \left(\frac{1}{H} \sum_i \sum_j \frac{GLRLM(i,j) i^2}{j^2}\right) \quad (27)$$

GLRLM_LRLGE, GLRLM_LRHGE: the distribution of the long homogeneous runs with low or high grey-levels.

$$GLRLM\_LRLGE = Average\ over\ 13\ (or\ 4)\ directions\ \left(\frac{1}{H} \sum_i \sum_j \frac{GLRLM(i,j) j^2}{i^2}\right) \quad (28)$$

$$GLRLM\_LRHGE = Average\ over\ 13\ (or\ 4)\ directions\ \left(\frac{1}{H} \sum_i \sum_j GLRLM(i,j) \cdot i^2 j^2\right) \quad (29)$$

GLRLM_GLNUr, GLRLM_RLNU: the non-uniformity of the grey-levels or the length of the homogeneous runs.

$$GLRLM\_GLNUr = Average\ over\ 13\ (or\ 4)\ directions\ \{\frac{1}{H}\sum_i(\sum_j \boldsymbol{GLRLM(i,j)})^2\} \quad (30)$$

$$GLRLM\_RLNU = Average\ over\ 13\ (or\ 4)\ directions\ \{\frac{1}{H}\sum_j(\sum_i \boldsymbol{GLRLM(i,j)})^2\} \quad (31)$$

GLRLM_RP: the homogeneity of the homogeneous runs.

$$GLRLM\_RP = Average\ over\ 13\ (or\ 4)\ directions\ \left(\frac{H}{\sum_i\sum_j\ (j\cdot GLRLM(i,j))}\right) \quad (32)$$

Table S5. Definition of radiomics features for Neighborhood Grey-Level Different Matrix (NGLDM).

NGLDM_Coarseness
NGLDM_Contrast
NGLDM_Busyness

The NGLDM corresponds to the difference of grey-level between one voxel and its 26 neighbours in 3 dimensions (8 in 2D). An element *(i,1)* of NGLDM corresponds to the probability of occurrence of level *i* and an element *(i,2)* is equal to:

$$NGLDM\ (i,2) = \sum_p\sum_q \begin{cases} |\bar{M}\ (p,q) - i| \sim if I(p,q=i) \\ 0 \sim else \end{cases} \quad (33)$$

where $\bar{M}(p,q)$ is the average of intensities over the 26 neighbor voxels of voxel*(p,q)*.

NGLDM_Coarseness: the level of spatial rate of change in intensity.

$$NGLDM\_Coarseness = \frac{1}{\sum_i NGLDM(i,1)\cdot NGLDM(i,2)} \quad (34)$$

NGLDM_Contrast: the intensity difference between neighboring regions.

$$NGLDM\_Contrast = \left[\sum_i\sum_j \boldsymbol{NGLDM(i,1)\cdot NGLDM(j,1)\cdot(i-j)^2}\right]\cdot\frac{\sum_i NGLDM(i,2)}{E\cdot G\cdot(G-1)} \quad (35)$$

where E corresponds to the number of voxels in the VOI and G the number of grey-levels.

NGLDM_Busyness: the spatial frequency of changes in intensity.

$$NGLDM\_Busyness = \frac{\sum_i NGLDM(i,1)\cdot NGLDM(i,2)}{\sum_i\sum_j |i\cdot NGLDM(i,1) - j\cdot NGLDM(j,1)|} \quad (36)$$
$$with\ NGLDM(i,1)\neq 0,\ NGLDM(j,1)\neq 0$$

Table S6. Definition of radiomics features for Grey-Level Zone Length Matrix (GLZLM).

GLZLM_SZE : Short-Zone Emphasis
GLZLM_LZE : Long-Zone Emphasis
GLZLM_LGZE : Low Gray-level Zone Emphasis
GLZLM_HGZE : High Gray-level Zone Emphasis
GLZLM_SZLGE : Short-Zone Low Gray-level Emphasis
GLZLM_SZHGE : Short-Zone High Gray-level Emphasis
GLZLM_LZLGE : Long-Zone Low Gray-level Emphasis
GLZLM_LZHGE : Long-Zone High Gray-level Emphasis
GLZLM_GLNUz : Gray-Level Non-Uniformity for zone
GLZLM_ZLNU : Zone Length Non-Uniformity is
GLZLM_ZP : Zone Percentage

The GLZLM provides information on the size of homogeneous zones for each grey-level in 3 dimensions. Element *(i,j)* of GLZLM corresponds to the number of homogeneous zones of *j* voxels with the intensity *i* in an image and is called *GLZLM(i,j)* thereafter.

GLZLM_SZE, GLZLM_LZE: the distribution of the short or the long homogeneous zones in an image.

$$GLZLM\_SZE = \frac{1}{H}\sum_i \sum_j \frac{GLZLM(i,j)}{j^2} \quad (37)$$

$$\text{GLZLM\_LZE} = \frac{1}{H}\sum_i \sum_j GLZLM(i,j) \cdot j^2 \quad (38)$$

where H corresponds to the number of homogeneous zones in the VOI.

GLZLM_LGZE, GLZLM_HGZE: the distribution of the low or high grey-level zones.

$$GLZLM\_LGZE = \frac{1}{H}\sum_i \sum_j \frac{GLZLM(i,j)}{i^2} \quad (39)$$

$$GLZLM\_HGZE = \frac{1}{H}\sum_i \sum_j GLZLM(i,j) \cdot i^2 \quad (40)$$

GLZLM_SZLGE, GLZLM_SZHGE: the distribution of the short homogeneous zones with low or high grey-levels.

$$GLZLM\_SZLGE = \frac{1}{H}\sum_i \sum_j \frac{GLZLM(i,j)}{i^2 j^2} \quad (41)$$

$$GLZLM\_SZHGE = \frac{1}{H}\sum_i \sum_j \frac{GLZLM(i,j) \cdot i^2}{j^2} \quad (42)$$

GLZLM_LZLGE, GLZLM_LZHGE: the distribution of the long homogeneous zones with low or high grey-levels.

$$GLZLM\_LZLGE = \frac{1}{H}\sum_i \sum_j \frac{GLZLM(i,j) \cdot j^2}{i^2} \quad (43)$$

$$GLZLM\_LZHGE = \frac{1}{H}\sum_i \sum_j GLZLM(i,j) \cdot i^2 \cdot j^2 \quad (44)$$

GLZLM_GLNUz, GLZLM_ZLNU: the non-uniformity of the grey-levels or the length of the homogeneous zones.

$$GLZLM\_GLNUz = \frac{1}{H}\sum_i \left(\sum_j GLZLM(i,j)\right)^2 \quad (45)$$

$$GLZLM\_ZLNU = \frac{1}{H}\sum_j \left(\sum_i GLZLM(i,j)\right)^2 \quad (46)$$

GLZLM_ZP: the homogeneity of the homogeneous zones.

$$GLZLM\_ZP = \frac{H}{\sum_i \sum_j (j \cdot GLZLM(i,j))} \quad (47)$$

Table S7. Intraclass correlation coefficient (ICC) results according to the each variables.

Variables	ICC	Low	Upper
CONVENTIONAL_SUVmin	0.9	0.82	0.95
CONVENTIONAL_SUVmean	0.93	0.87	0.96
CONVENTIONAL_SUVstd	0.93	0.86	0.96
CONVENTIONAL_SUVmax	0.9	0.82	0.95
CONVENTIONAL_SUVpeak	0.98	0.96	0.99
CONVENTIONAL_TLG	0.99	0.98	0.99
HISTO_Skewness	0.78	0.59	0.88
HISTO_Kurtosis	0.96	0.92	0.98
HISTO_Entropy_log$_{10}$	0.94	0.9	0.97
HISTO_Entropy_log$_2$	0.94	0.9	0.97
HISTO_Energy	0.91	0.83	0.95
SHAPE_Volume_mL	0.99	0.98	0.99
SHAPE_Volume_vx	0.98	0.97	0.99
SHAPE_Sphericity	0.97	0.95	0.98
SHAPE_Compacity	0.99	0.98	0.99
GLCM_Homogeneity	0.85	0.73	0.92
GLCM_Energy	0.78	0.59	0.88
GLCM_Contrast	0.86	0.74	0.92
GLCM_Correlation	0.99	0.98	0.99
GLCM_Entropy_log$_{10}$	0.95	0.91	0.97
GLCM_Entropy_log$_2$	0.95	0.91	0.97
GLCM_Dissimilarity	0.86	0.75	0.93
GLRLM_SRE	0.94	0.89	0.97
GLRLM_LRE	0.93	0.87	0.96
GLRLM_LGRE	0.96	0.93	0.98
GLRLM_HGRE	0.93	0.87	0.96
GLRLM_SRLGE	0.95	0.92	0.98
GLRLM_SRHGE	0.93	0.86	0.96
GLRLM_LRLGE	0.95	0.9	0.97
GLRLM_LRHGE	0.96	0.92	0.98
GLRLM_GLNU	0.97	0.95	0.99
GLRLM_RLNU	0.99	0.98	0.99
GLRLM_RP	0.93	0.87	0.95
NGLDM_Coarseness	0.98	0.96	0.99
NGLDM_Contrast	0.85	0.72	0.96
NGLDM_Busyness	0.93	0.88	0.96
GLZLM_SZE	0.95	0.91	0.97
GLZLM_LZE	0.94	0.89	0.97
GLZLM_LGZE	0.94	0.9	0.97
GLZLM_HGZE	0.92	0.85	0.96
GLZLM_SZLGE	0.89	0.79	0.94
GLZLM_SZHGE	0.89	0.81	0.94
GLZLM_LZLGE	0.94	0.89	0.97
GLZLM_LZHGE	0.95	0.92	0.98
GLZLM_GLNU	0.99	0.98	0.99
GLZLM_ZLNU	0.99	0.98	0.99
GLZLM_ZP	0.92	0.86	0.96

The inter-observer agreement of feature extraction was evaluated by using an intraclass correlation coefficient (ICC). The strength of agreement was evaluated as follows: an ICC value of 0.81–1.0, excellent agreement; 0.61–0.80, good agreement; 0.41–0.60, moderate agreement; 0.21–0.40, fair agreement and less than 0.20 indicated poor agreement.
